# Towards applying the essential public health functions for building health systems resilience: A renewed list and key enablers for operationalization

**DOI:** 10.3389/fpubh.2022.1107192

**Published:** 2023-01-20

**Authors:** Yu Zhang, Geraldine McDarby, Redda Seifeldin, Saqif Mustafa, Suraya Dalil, Gerard Schmets, Natasha Azzopardi-Muscat, James Fitzgerald, Awad Mataria, Ernesto Bascolo, Sohel Saikat

**Affiliations:** ^1^World Health Organization, Geneva, Switzerland; ^2^WHO Regional Office for Europe, Copenhagen, Denmark; ^3^Pan American Health Organization, Washington, DC, United States; ^4^WHO Regional Office for the Eastern Mediterranean, Cairo, Egypt

**Keywords:** essential public health functions, public health, health system resilience, universal health coverage (UHC), health system strengthening (HSS), population health needs

## Abstract

The COVID-19 pandemic, climate change-related events, protracted conflicts, economic stressors and other health challenges, call for strong public health orientation and leadership in health system strengthening and policies. Applying the essential public health functions (EPHFs) represents a holistic operational approach to public health, which is considered to be an integrated, sustainable, and cost-effective means for supporting universal health coverage, health security and improved population health and wellbeing. As a core component of the Primary Health Care (PHC) Operational Framework, EPHFs also support the continuum of health services from health promotion and protection, disease prevention to treatment, rehabilitation, and palliative services. Comprehensive delivery of EPHFs through PHC-oriented health systems with multisectoral participation is therefore vital to meet population health needs, tackle public health threats and build resilience. In this perspective, we present a renewed EPHF list consisting of twelve functions as a reference to foster country-level operationalisation, based on available authoritative lists and global practices. EPHFs are presented as a conceptual bridge between prevailing siloed efforts in health systems and allied sectors. We also highlight key enablers to support effective implementation of EPHFs, including high-level political commitment, clear national structures for institutional stewardship on EPHFs, multisectoral accountability and systematic assessment. As countries seek to transform health systems in the context of recovery from COVID-19 and other public health emergencies, the renewed EPHF list and enablers can inform public health reform, PHC strengthening, and more integrated recovery efforts to build resilient health systems capable of managing complex health challenges for all people.

## Introduction

The health, social and economic costs of health systems shocks continue to underscore the need for more focus on public health (1, 2). Despite bringing high returns on social and health investments (3–5), public health has often been obscure in planning and accorded low priority, limited political support and inadequate funding. As many countries move from the acute phase of the COVID-19 response after over 2 years of the pandemic, governments are planning for socioeconomic recovery in the context of likely fiscal pressure. To ensure sustainable impact and efficiency from investments, global and national policies must put public health and health systems at the heart of recovery efforts by addressing critical gaps in health systems foundations and strengthening multisectoral collaboration for health.

The essential public health functions (EPHF) are a set of fundamental and interconnected activities and capacities both within and beyond the health sector, required to ensure effective public health actions (6–8). Pre-COVID-19, strengthening EPHFs to ensure global health security, universal health coverage (UHC) and greater health equity was a key recommendation in several health resolutions and declarations (9–11). The Declaration of Astana in 2018 affirmed world leaders' commitment to strengthen primary health care (PHC) towards UHC (10); the Operational Framework for Primary Health Care provides support to achieve the goals and objectives of the declaration (12). The framework highlights EPHFs as core to meeting population health needs (12). While there has been an increased momentum in application of EPHFs for health systems resilience and UHC, for example, in the Americas since 2020 (13), historically, EPHFs have been utilized primarily in national public health capacity assessments (14–16), health workforce planning and education (17), and the development of public health institutes (18), with limited systematic application in health system strengthening (8). Their impact as an integrated approach to strengthening public health capacities at national, subnational and service delivery levels including primary care, and bridging programmes and sectors for health systems resilience has been undermined by a failure to operationalise their interconnectedness, together with the lack of an up-to-date unified list to facilitate global consensus on defining the operational scope of and catalyzing meaningful investments in public health. When sufficiently resourced and applied holistically, EPHFs can provide an operational approach to promoting and protecting individual and population health that is both sustainable and affordable (9).

The unprecedented scale of the impacts of COVID-19 has demonstrated that traditional and siloed approaches to health systems, including traditional health system strengthening focused on clinical services, vertical programmes, health security programmes and humanitarian responses (Supplementary Figure 1), while providing dedicated focus and short-term visibility of impact, have failed to achieve the long-term system strengthening required to attain efficiency, optimize health outcomes and maintain services during shock events (19–22). This has brought a renewed focus to EPHFs, with global and national actors reviewing their performance and seeking a recovery that builds health systems capable of preventing, responding to and learning from evolving health challenges including emergencies (13, 23–25). In this context, this perspective article, based on a WHO discussion paper published early this year which synthesized the best available global evidence on operationalizing EPHFs (8), informs a comprehensive and integrated approach to EPHFs through a renewed list of EPHFs and the identification of key enablers for effective operationalization. These can inform national health authorities and global actors that provide country support (e.g., WHO, international donors, intergovernmental organizations) in health system strengthening, reform and recovery that promotes resilience.

## Essential public health functions – a renewed global reference list

The concept of EPHFs emerged in the context of a rapidly changing health, social and political landscape in countries worldwide in late twenteeth century (7, 26, 27). Since the 1980s, EPHFs were developed in the Americas to define fundamental State functions for efficient and effective public health programmes; this responded to the need to strengthen health authorities' stewardship role in the context of weakening public health in health sector reforms (7, 27). At the same time in Eurasia, the newly independent states of the former Soviet Union experienced dramatic system changes and health consequences, and many other countries also experienced fast shifts in epidemiological and demographic landscapes. There was a demand to identify a set of essential functions (i.e., EPHFs) to ensure public health systems could function and deliver public health services in an optimal way to respond to emerging and priority population health needs (7, 26).

The EPHF list defined by WHO through a Delphi exercise in 1997 represented the first global reference against which countries could benchmark their public health capacities (26, 27). Since then, several global health actors and national health authorities^1^ have developed their own lists and approaches, including assessments of EPHFs to identify gaps in technical capacity or inform country-focused support. After entering the twenty first century, global experience with public health emergencies (e.g., SARS, MERS, Ebola, Zika, and COVID-19) and other emerging health issues (e.g., increasing burden of noncommunicable diseases, rising antimicrobial resistance threats and environmental hazards) has continued to reveal insufficient baseline public health capacities and the lack of an integrated approach to managing the wide range of public health challenges (8). This necessitates a re-examination of existing EPHF lists to ensure they reflect the present understanding of public health and evolving population health needs, while also reflecting the different dimensions and scope of various approaches to the application of EPHFs. The resultant unified list can provide a focal point to draw the required attention from decision makers globally to influence the direction of national priority setting.

A crosswalk analysis of existing authoritative lists^2^ was conducted and results were presented in the discussion paper “*Twenty first century health challenges: can the essential public health functions make a difference?”* (8). Findings indicate a consensus on the fundamental operational remit of public health which formed the basis of developing the new consolidated list of 12 EPHFs (Box 1) (8). This list consists of activities commonly recognized as essential, such as monitoring, evaluation and surveillance, public health emergency management, health promotion, and disease prevention. It also contains activities underrepresented in earlier lists that are increasingly recognized as necessary to meet population health needs, such as the rational and equitable use of health technologies and the public health workforce (8, 27).

Box 1 **A consolidated list of EPHFs (adapted from the WHO discussion paper) (8).^3^**

Monitoring and evaluating population health status, health service utilization and surveillance of risk factors and threats to health *(public health intelligence)*

Managing public health emergency *(emergency management)*

Assuring effective public health governance, regulation, and legislation *(public health governance)*

Supporting efficient and effective health systems and multisectoral planning, financing and management for population health *(public health planning and financing)*

Protecting populations against health threats, including environment and occupational hazards, communicable disease threats, food safety, chemical and radiation hazards *(health protection)*

Promoting prevention and early detection of communicable and noncommunicable diseases *(disease prevention and early detection)*

Promoting health and well-being and actions to address the wider determinants of health and inequity *(health promotion)*

Ensuring community engagement, participation and social mobilization for health and well-being *(communication participation)*

Ensuring adequate quantity and quality of public health workforce (public health workforce)

Assuring quality of and access to health services *(quality and access)*

Advancing public health research *(research)*

Ensuring equitable access to and rational use of essential medicines and other health technologies *(equal and safe access to medical products)*

**Figure 1:**
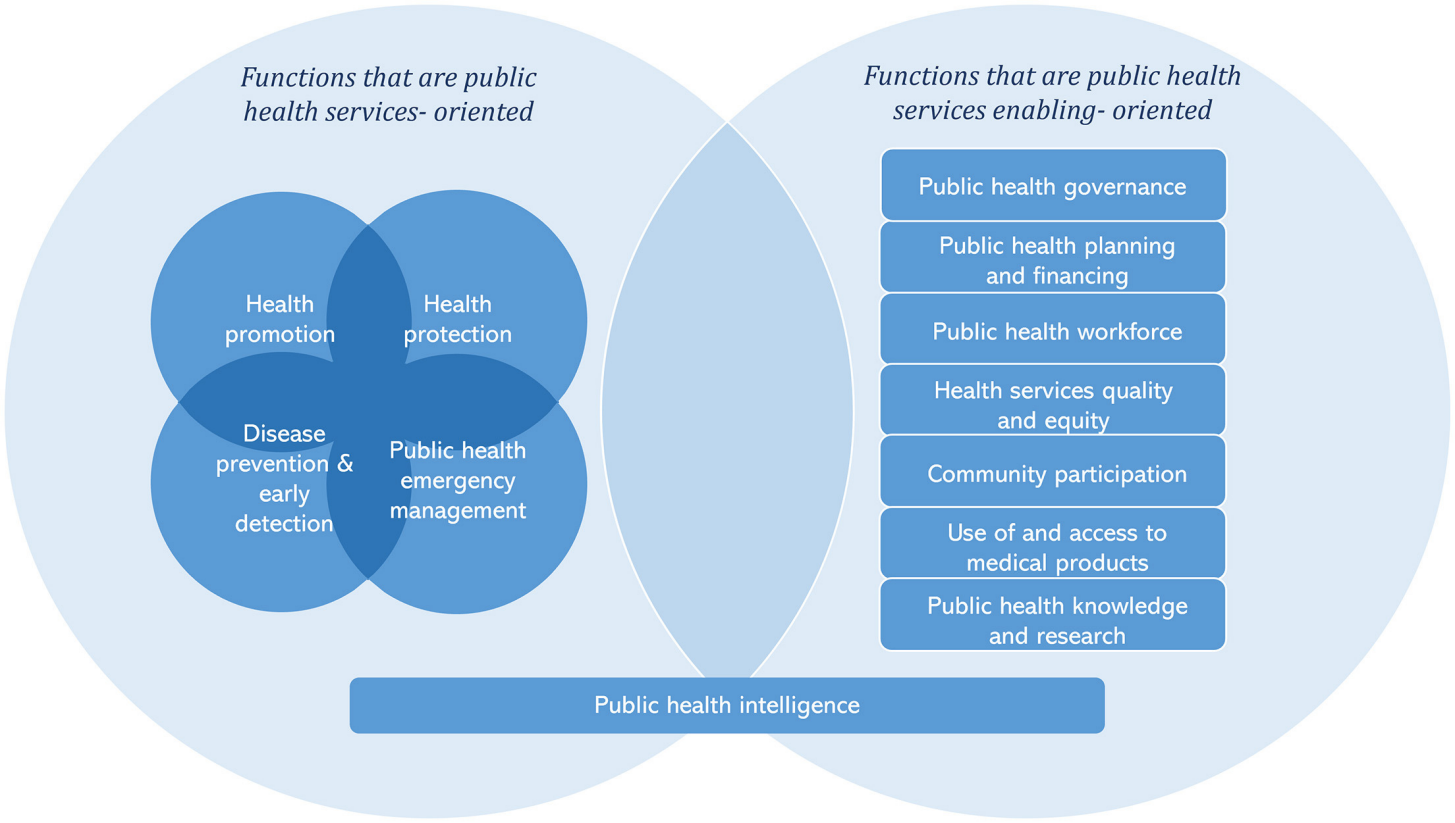
Interlinked essential public health functions working together to provide public health services to the populations.

While all EPHFs contain both service delivery and enabling elements, to inform operationalisation, the EPHFs can be further grouped according to activities that are primarily service focused and those that essentially enable the delivery of public health services (Figure 1). This differentiation is based on the experience of applying EPHFs in different regions (15, 16, 27). The service-oriented activities include promotive, preventive, and protective public health services for populations that should be integrated into service delivery platforms at all levels including a focus on primary care. The enabling activities include activities embedded in health systems, communities and beyond the health sector required to foster and facilitate the delivery of public health services. Public health intelligence is a crosscutting activity that is both service oriented and has an enabling characteristic. By identifying all activities required for effective public health practice, the consolidated list of EPHFs can serve as a renewed global reference for countries reforming their national public health architecture and capacities as part of recovery from the COVID-19 pandemic and other shock events.

## Operationalising the essential public health functions - key enablers for integrated health system strengthening unpinned by public health

There are various entry points into health systems for policies, planning and investments, including disease and life course focused efforts and emergency response and humanitarian efforts. However, these traditional routes have not developed public health capacities sufficiently or comprehensively enough to ensure health systems resilience. EPHFs represent an integrated approach to health system strengthening across the multiple entry points to health systems, providing a bridge between these efforts in policies, planning, implementation and assessment (Box 2). Focusing on the EPHFs and primary health care as the foundation for health systems strengthening supports the objectives of the individual programmes while also contributing to health system resilience and broader health goals including equity and efficiency.

Box 2 **An overview of the EPHFs as a bridge between global efforts to promote UHC and health security.**

A further comparative analysis of the updated EPHF list against a number of global frameworks^4^ was conducted to assess the potential of EPHFs to provide conceptual bridging between approaches to achieve UHC and health security (8). A portion of this analysis, presented below, indicates complementarity across these agendas, highlighting opportunities where investment in EPHFs strengthens health systems and promotes health security in tandem (Supplementary Figure 2).

For example, the Primary Health Care Operational Framework identifies 14 levers that are required to accelerate progress in strengthening PHC-oriented systems and advancing UHC (12); the IHR [2005] defines core capacities required to detect, assess, notify and report events and respond to public health risks and emergencies of national and international concern (28). Investing in EPHFs can recognize and strengthen the role of PHC in emergency preparedness and response, supporting health security by strengthening PHC based surveillance, triaging and case management. Strengthening emergency surveillance and response capacities, as a public health function, meets IHR requirements and helps to reduce the burden on secondary and tertiary care during public health events, promoting resilience. This highlights the potential role of EPHFs in bridging the currently siloed efforts towards achieving these interdependent global health targets, and building more integrative, holistic and equitable health systems.

While the EPHF list provides a foundation, the use of EPHFs as an operational approach to integrated health system strengthening requires actions across specific areas or enablers. In the discussion paper “*Twenty first century health challenges: can the essential public health functions make a difference?”*, several interconnected enablers, which are recurrent themes in literatures and based on a review of available global experience with EPHFs, were identified as necessary to ensure adequate investment in and delivery of EPHFs (8). In this section, we further discuss and expand on three key enablers: high-level political commitment to public health with EPHFs, multisectoral accountability mechanisms for delivering EPHFs, and assessment of EPHF provision.

### High-level political commitment to strengthen public health with the essential public health functions

Several reviews have highlighted the critical role of political commitment in determining the success or failure of public health initiatives, from implementation of the IHR (2005) (29) to vector-borne disease elimination (30). Strengthening public health with EPHFs also requires sustained high-level political commitment to ensure long term health sector and intersectoral actions that optimize population health (8). This can be a challenge when governments are driven by short-term wins while returns on public health investment tend to be less visible or seem more long term by comparison (31, 32).

Political commitment can be solidified through including EPHFs in health legislation, prioritizing EPHFs within health policies, strategies and plans; allocating dedicated funding to EPHFs in multi-year budgets; establishing clear governance structures to lead, coordinate and oversee the delivery of EPHFs, etc.

One of the approaches to solidifying political commitment to strengthening public health can be establishing or capacitating a national public health institute (NPHI). An NPHI is a government organization or a network of organizations that are science-based and provide national leadership and coordination of public health efforts to improve population health outcomes (18, 33–35). EPHFs provide guidance for defining the scope and functions of NPHIs (18, 36, 37). Organizing public health leadership and expertise within an NPHI can support to improve the efficiency of the implementation of public health functions (including health security) and improve public health stewardship and accountability (36, 38–40).

Informed by lessons learned from recent public health events, several countries have established or reformed NPHIs to provide the oversight of a number of EPHFs (if not all of these functions) (35–37, 41). This has often been in response to acute health threats or enduring public health challenges as well as the growing need to consolidate public health functions under one roof (33, 35–37). For example, with the demand to enhance commitment and leadership of the EPHFs under a single focal point to respond to multifaceted public health threats, Kenya National Public Health Institute was established to bring together EPHFs from across the government and health system, following the *Kenya National Public Health Institute Order, 2021* (42, 43). To be effective NPHIs need to be capacitated with adequate visibility, authority, independence, legitimacy, and resources, and supported by structures at subnational levels (8, 35, 45, 46). This further strengthens the stewardship role of health authorities in planning and oversight of EPHFs, which span health and allied sectors (referring to stakeholders in public health outside Ministry of Health, such as environment; food and road safety; urban planning; and local authority services), from national to community levels (27).

Global and regional networks and cooperative bodies, such as the International Association of National Public Health Institutes, Africa Centers for Disease Control and Prevention, the European Centers for Disease Control and Prevention have been supporting countries to foster a coherent approach to building public health capacities with strong stewardship for EPHFs, including developing or strengthening NPHIs towards a more systems-oriented role that is reflective and responsive to the growing and evolving profile of global health challenges (27, 37).

### Multisectoral accountability mechanism for delivering the essential public health functions

The broader determinants of health (including social, behavioral, environmental, commercial determinants) and multisectoral nature of public health actions necessitate an approach to public health that goes beyond health systems. Such a public health approach would support intersectoral planning, budgeting and actions to address determinants of health. The EPHFs promote a whole of government and whole of society orientation towards health and wellbeing. The PHC approach also requires governments at all levels to enable actions and accountability beyond the health sector to deliver the EPHFs needed to meet population health needs in peacetime and during emergencies.

Accountability is a matter of knowing and agreeing; acting and being responsible; being answerable; and reporting and monitoring (47–49).

A multisectoral approach to accountability provides a means to define the commitments and actions that governmental and non-governmental entities within and beyond the health sector are accountable for and how they might be held accountable within public health agendas (50, 51). This is to ensure intersectoral action for health, which is recognized as essential to support health and wellbeing but is often hindered by a lack of adequate accountability mechanisms to support implementation (27, 52–54).

Establishing multisectoral accountability mechanisms for EPHFs can learn from experiences in existing multisectoral coordination mechanisms in specific areas including One Health platforms. Informed by lessons learnt from the 2014–2016 Ebola crisis, Guinea, Liberia, and Sierra Leone established their national One Health platform structures anchored in the offices of the state head, inter-ministerial committees, or ministries of health; these platforms have facilitated development of national intersectoral action plans for antimicrobial resistance, zoonotic diseases, etc (55). Coordinating efforts through One Health mechanisms can improve resource efficiency (55), though national One Health mechanisms often need strengthening including broadening stakeholder representations (56, 57), the addition of a solid monitoring and evaluation component (56), and integrated structures for different One Health areas (58).

A recent effort at the international level towards fostering multisectoral accountability for EPHFs in countries involves a global roadmap to build an integrated and multisectoral public health workforce to implement EPHFs. This roadmap recognizes that various occupations in health and allied sectors deliver EPHFs and calls for mapping, measurement and development of this workforce across sectors (59, 60).

### Systematic assessment of delivery of the essential public health functions

The systematic assessment of EPHF delivery identifies baseline public health capacities and areas for improvement (8). There are a number of self-assessment tools that focus on the evaluation of each public health function and its sub-functions through stakeholder workshops and scoring with national and external stakeholders (15, 16). Most recently, Armenia (61) and Slovenia (62) conducted self-assessment of EPHFs, which identified strengths and priority areas for improvement in public health capacities and services and formed recommendations to stakeholders. Experiences of joint EPHF self-assessment in the Americas, Central Asia, Europe, Middle East and North Africa showed that this approach contributed to evidence based priority selection for public health reform and promoted a greater intersectoral understanding of public health but can be somewhat unwieldly and follow-up actions to implement recommendations are not well documented (27, 63–65).

A new approach recently developed in collaboration between WHO and Ireland focuses instead on assessing EPHFs as a whole and at a strategic level (Box 3). Another recent example of high-level assessment is the integration of EPHFs in to primary health care measurement frameworks to monitor institutional capacity to deliver EPHFs (66). In addition, building the capacity of health information systems to incorporate and collect data from a population perspective and leveraging existing information systems can also enhance monitoring and evaluation of EPHFs. Routine health information systems such as District Health Information Software (67, 68) provide rich information on population health needs (which supports the prioritization of public health action), performance of public health system and programmes (which are public health functions themselves), and population health outcomes (which reflects the effectiveness of EPHFs implicitly). Utilizing the results of assessments to build institutional capacities is crucial to support health systems resilience for public health (27, 64).

Box 3 **Assessing EPHFs to strengthen public health stewardship and capacities.**

In January 2022, Ireland embarked on a reform process with the aim of strengthening public health capacities in light of national and international lessons identified from experience with COVID-19 (44). A thematic approach to assessment using the EPHFs as a lens, was used to review the current delivery of public health with respect to policy and planning, infrastructure, service delivery and integration and coordination (69). The resultant analysis was used to identify strengths to be leveraged and actionable policy options to optimize the delivery of public health through improved stewardship and operationalisation of EPHFs (69). The findings of the analysis have been used to support high level advocacy to support the strategic shift towards public health needed to ensure resilience. Ireland is now using the EPHFs to define the operational scope of public health in Ireland and to identify the scope and functions of a new national public health institute.

## Conclusion

COVID-19 and other public health challenges have repeatedly proven that health systems are vital for social and economic stability and development. Years of underinvestment and lack of a comprehensive public health approach to strengthening health and allied systems have had significant consequences. The majority of countries lack sufficient public health capacities for effective prevention, early warning and case management, and have struggled to maintain essential health services while responding to COVID-19. In addition, countries continue to face other public health challenges, such as noncommunicable diseases, antimicrobial resistance, climate change, an aging population and health inequity, that place increasing demands on already struggling health systems.

The case for investment in public health capacities and institutions is increasingly clear (4, 32, 70). The current political and public impetus for public health, resulting from the global experience with COVID-19, represents a brief, yet valuable opportunity for countries to rethink their approach to investing in public health for building health systems resilience. EPHFs can serve as a holistic and integrated approach to enhancing public health capacities within and beyond health systems. Lessons identified from COVID-19 also highlight that the way PHC changes, adapts, and re-designs its organization to respond to the needs of the population is key to effective response to infectious disease outbreaks (71–74). The EPHFs can be utilized to strengthen primary health care by supporting planning and holistic integration of public health services to primary care to constitute integrated health services as outlined in the operational framework for primary health care. In many countries, primary care is often the first contact point with the health systems. Improving integration of public health and primary care benefits individuals as well as wider populations. Further work is needed to delineate an essential package of public health services as part of integrated health services to be delivered at primary care level from the EPHFs lens.

**Figure 2:**
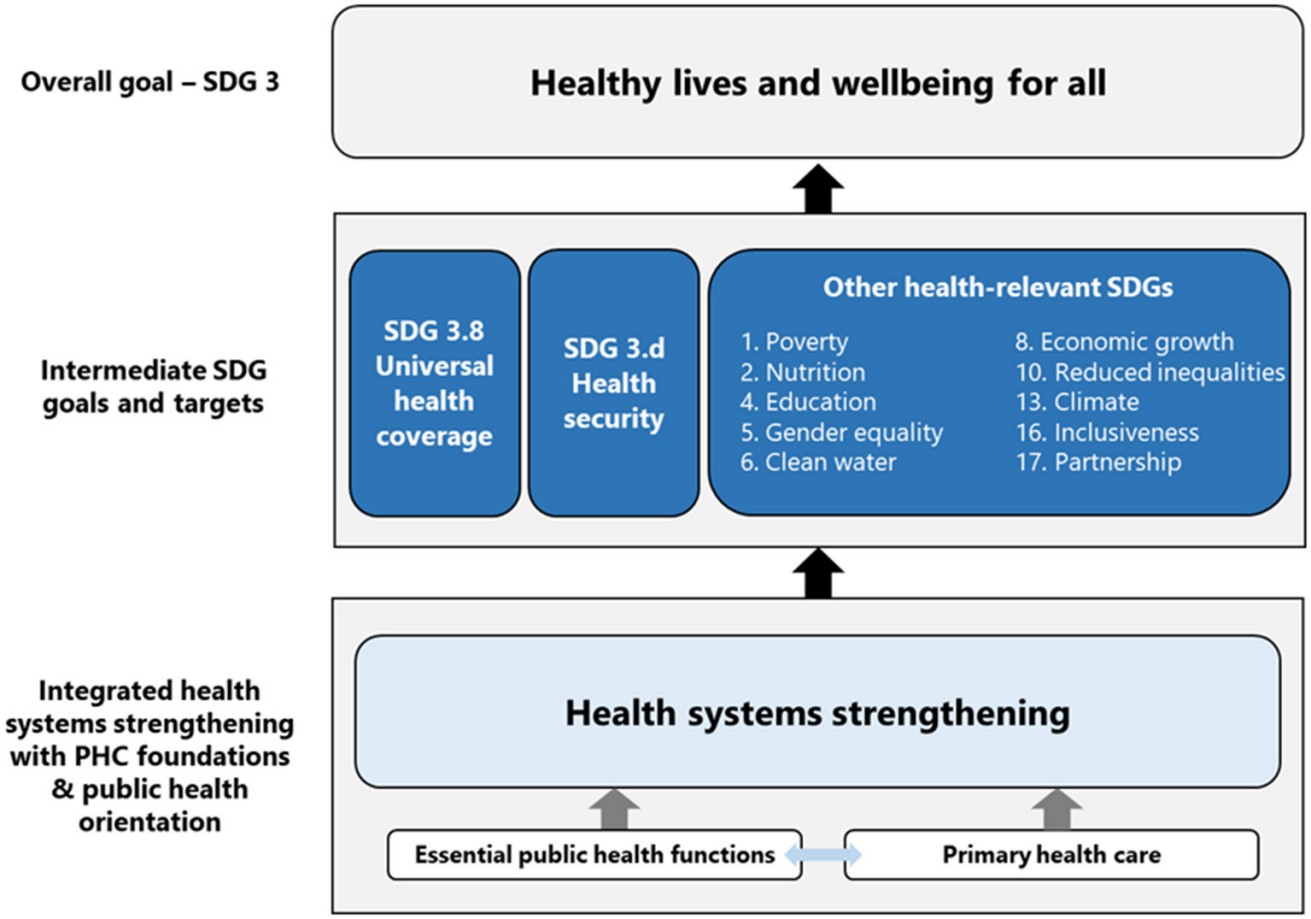
Illustration how health systems strengthening with primary health care foundations and public health orientation contributes to SDG 3 through supporting UHC, health security and other health-related SDG targets [adapted from (77, 78)].

The COVID-19 pandemic is threatening years of progress in global health as backward sliding of the Sustainable Development Goals (SDG) targets has been seen or predicted. COVID-19 reversed the progress made in the fight against tuberculosis, with a 19% drop in number of people treated for drug-resistant tuberculosis in 2020 compared to in 2019 (75). COVID-19 also caused widespread disruption of essential health services with 92% of the countries still reporting service disruption in late 2021; this is likely to halt the progress made towards UHC which had already fallen behind reaching SDG target 3.8 in pre-COVID times (21, 76). Countries need to reaffirm the commitment to reaching SDGs by 2030. In this context, complementary to primary health care, the EPHFs can support countries in strengthening health systems foundations that are public health oriented for UHC, health-related SDGs and health security (Figure 2).

In this perspective article, we proposed several key enablers for applying EPHFs to strengthen health system with strong public health orientation. One of the limitations is grounded in the fact that there are limited resources documenting the application of EPHFs to decision-making and high-level policies and planning in countries, besides EPHF assessment. As more national authorities in Europe, Americas, the Middle East, etc. are utilizing EPHFs or planning to embark on applying EPHFs, we will be able to learn from their experiences. With the consolidated EPHF list as a reference, countries need to secure political commitment to public health and a more integrated approach to health systems strengthening unpinned by EPHFs; reform government structures to ensure clear public health leadership and coordination of intersectoral action for health; strengthen multisectoral accountability for delivering EPHFs; and assess the current state of EPHF stewardship and provision. Action on these interconnected enablers within countries can facilitate greater efficiency, effectiveness and equity in addressing the complex public health challenges of today.

## Data availability statement

The original contributions presented in the study are included in the article/Supplementary material, further inquiries can be directed to the corresponding author.

## Author contributions

SS, YZ, and GM contributed to the conception and design of the research. YZ and GM collected information and led the analysis. YZ, GM, RS, SM, and SS developed the first draft of the manuscript. SD, GS, NA-M, JF, AM, and EB reviewed the manuscript and provided inputs to reflect global and regional practices. SS coordinated inputs from all authors. All authors read and approved the submitted version.

## Footnotes

^1^This includes a number of WHO regional offices, the World Bank, Brazil, Canada, China, the United States, etc.

^2^The analysis included lists of EPHFs or equivalent frameworks developed by the Centers for Disease Control and Prevention of the United States in 1994, 2012 and 2020; the WHO in 1998; the National Public Health Partnership of Australia in 2000; the Pan American Health Organization in 2001 and 2020; the National Health Service of the United Kingdom in 2001; the WHO Regional Office for the Western Pacific in 2003; the World Bank in 2004; the Ministry of Health of Indonesia in 2004; the Ministry of Health of British Columbia in 2005; the Ministry of Health of Brazil in 2006; the Public Health Clinical Network of New Zealand in 2011; the Israeli Association of Public Health Physicians in 2012; the European Commission in 2014; the WHO Regional Office for Europe in 2014; the National Health Commission of China in 2015; and the WHO Regional Office for the Eastern Mediterranean in 2017.

^3^The texts in brackets represent short names of each function in Box 1.

^4^These frameworks include health system building blocks, the primary health care operational framework, the International Health Regulations (IHR 2005) core capacities and health emergency and disaster risk management framework.
