# Supplementary material for: Towards applying the essential public health functions for building health systems resilience: A renewed list and key enablers for operationalization
Source: Front Public Health. 2023 Jan 20;10:1107192. doi: 10.3389/fpubh.2022.1107192 (PMC9895390; doi:10.3389/fpubh.2022.1107192)
Supplement: Supplementary file 1 [file Data_Sheet_1.docx]

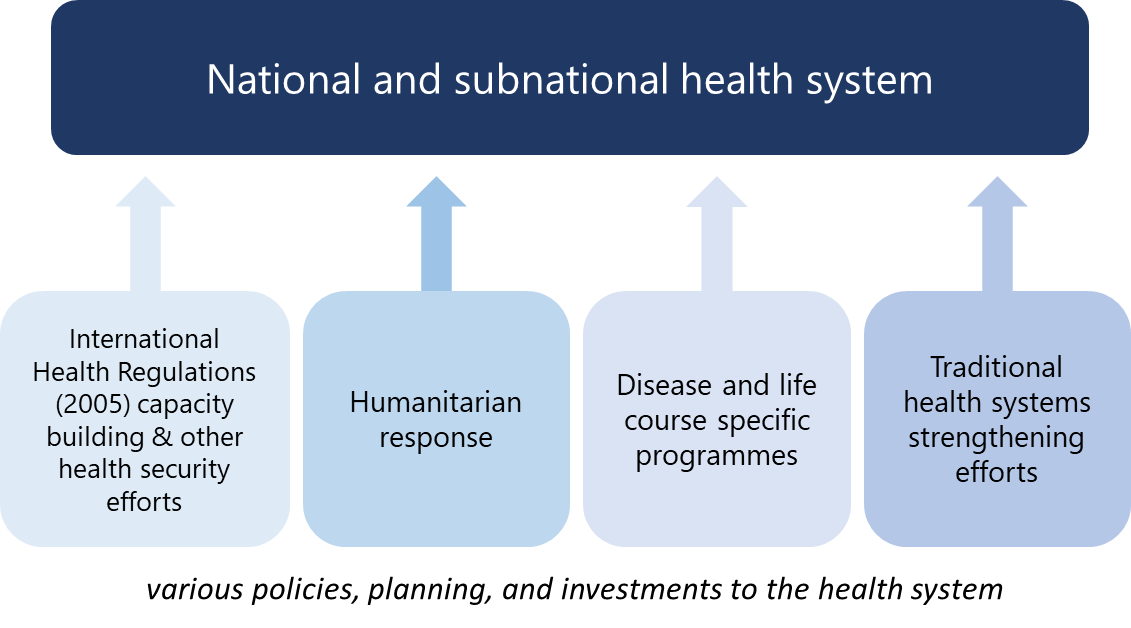


Fig. S1. An example illustration of health system fragmentation - multiple parallel entries into a national health system


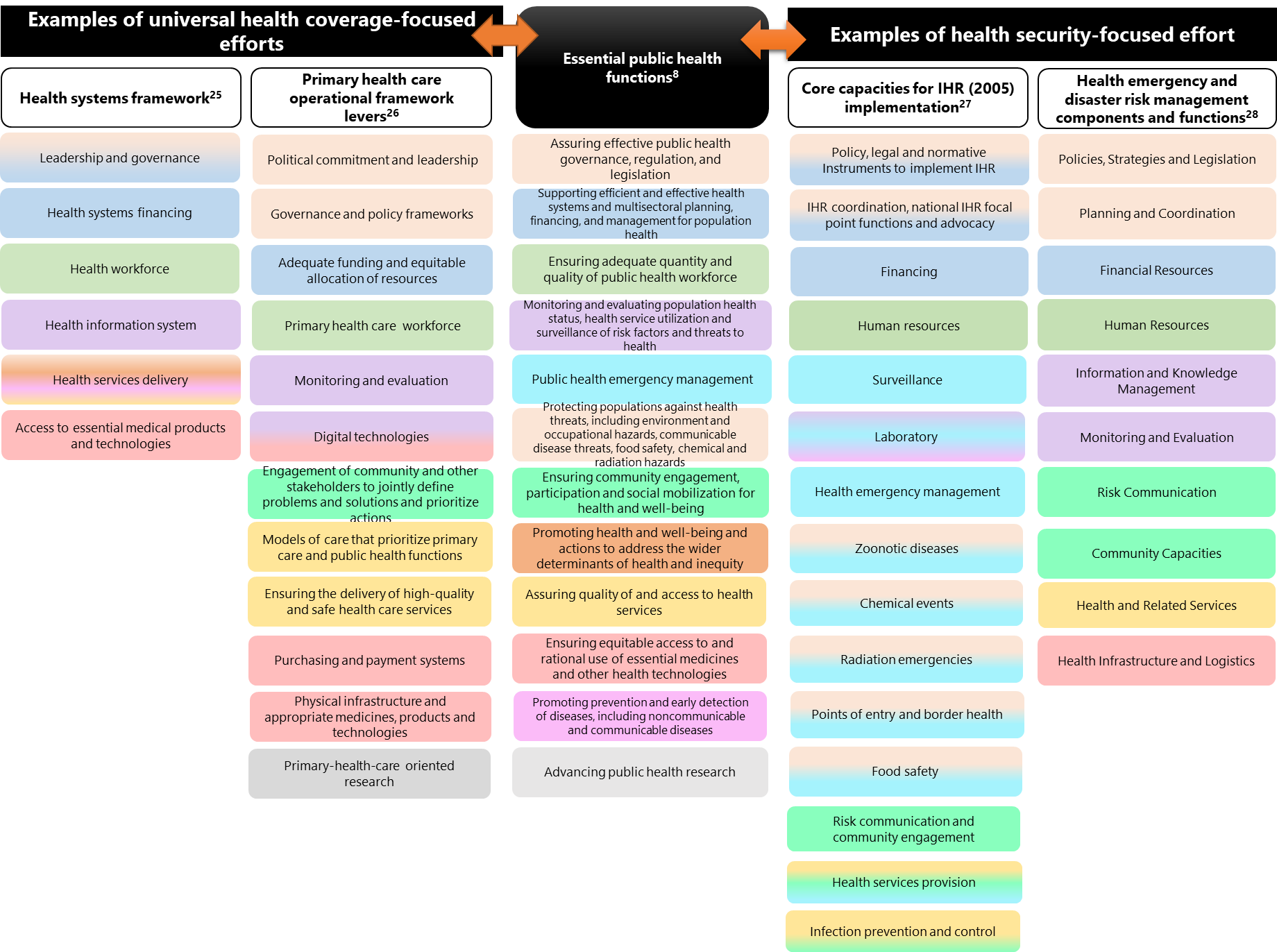


Note: the same color across different frameworks stands for conceptual similarity.

Fig. S2. Crosswalk analysis of EPHFs, health system building blocks, core capacities required by IHR (2005), primary health care operational framework, and health emergency and disaster risk management framework.
